# Food Sustainability Knowledge and Attitudes in the Spanish Adult Population: A Cross-Sectional Study

**DOI:** 10.3390/nu12103154

**Published:** 2020-10-15

**Authors:** Ángela García-González, María Achón, Alejandra Carretero Krug, Gregorio Varela-Moreiras, Elena Alonso-Aperte

**Affiliations:** 1 Departamento de Ciencias Farmacéuticas y de la Salud, Facultad de Farmacia, Universidad San Pablo-CEU, CEU Universities, Urbanización Montepríncipe, Boadilla del Monte, 28660 Madrid, Spain; achontu@ceu.es (M.A.); alejandra.carreterokrug@ceu.es (A.C.K.); gvarela@ceu.es (G.V.-M.); eaperte@ceu.es (E.A.-A.); 2 Spanish Nutrition Foundation (FEN), 28024 Madrid, Spain

**Keywords:** sustainable food, sustainable consumer behavior, food knowledge and attitudes, sustainable eating

## Abstract

Moving towards sustainable food systems and food consumption are proposed as strategies to reduce environmental impact. However, these strategies require joint action between different stakeholders, including the general population’s knowledge and perception, as final consumers. To assess the knowledge and awareness on food sustainability and environmental impact concepts in a representative sample of Spanish adult population, we conducted a cross-sectional, nationally representative telephone survey. After random selection, 2052 respondents aged ≥18 years (57% woman and 43% men) participated. A questionnaire was specifically designed for the research. Participants showed a good attitude towards sustainable diets, which were described as healthy by 40% of the population. Most of the responders (>70%), did not well understand ecological and carbon footprints, and green and blue water concepts. Men declared a higher understanding of sustainability concepts, as compared to women. More than 50% of the population misunderstood the impact of meat and derivatives production on sustainability, and 70% perceived the same for fish and dairy products. Women were more prone to pay more money to afford a sustainable diet than men were. In conclusion, although consumers show a positive attitude towards sustainability, important misconceptions remain, and thus require intervention through education, information, and motivation.

## 1. Introduction

The Intergovernmental Panel on Climate Change (IPCC), the group of the United Nations responsible for assessing scientific knowledge related to climate change, has very recently stated that climate change has to be challenged through a global approach where sustainability is the main value [1]. Footprint analyses show that humanity’s ecological footprint is 1.7 times the Earths, a metric that divides the Ecological Footprint by the global biocapacity available to each person in the world [2]. The implementation of coordinated initiatives to deal with climate change can involve the simultaneous improvement of land, food security, and nutrition, as well as helping to end hunger. The already mentioned report also shows that approximately one third of the food produced is spoiled or wasted. The reasons why food is wasted are substantially different between developed and developing countries. Reducing food loss and waste would result in a decrease in greenhouse gas emissions and would help to improve food security [1].

Balanced diets based on plant foods (such as grains, legumes, fruits, and vegetables), and sustainably produced animal foods (i.e., in systems that generate few greenhouse gas emissions), provide greater opportunities for adaptation to climate change and limitation of its effects [1]. Specifically, sustainable diets are defined by the Food and Agricultural Organization (FAO) as “those diets with low environmental impacts which contribute to food and nutrition security and to healthy life for present and future generations. Sustainable diets are protective and respectful of biodiversity and ecosystems, culturally acceptable, accessible, economically fair and affordable, nutritionally adequate, safe, and healthy; while optimizing natural and human resources” [3].

Shifting food systems towards greater sustainability requires joint action from farmers, food industry, consumers, academia, nutrition science, stakeholders, and policy makers. As recently reviewed by Berry (2019) [4], the resulting synthesis has been discussed in three summary publications: the Lancet EAT-Lancet ommission on healthy diets from sustainable food systems [5]; the international expert report on the policy reform and realignment that is required to build sustainable food systems in Europe [6]; and the FAO report on biodiversity for food and agriculture [7].

The Mediterranean Diet has been traditionally selected by FAO as a model for the assessment of the sustainability of diets [4]. It has been scientifically well characterized and recognized as a healthy dietary pattern, and a greater adherence to the Mediterranean diet has been widely associated with significant improvements in health status. Moreover, Mediterranean Diet has also been analyzed in many surveys and appreciated for its low environmental impact [4,8,9]. However, despite the well-documented health and environmental benefits of the Mediterranean Diet, current data show a decline in adherence in the Mediterranean area, including Spain, due to multifactorial influences, such as life styles changes, food globalization, economic, and socio-cultural factors [4,10,11]. Loosing adherence has consequences, among others, on sustainability of food dietary patterns. 

The most well-known practical actions towards sustainable food systems are some strategies that have been promoted by different organisms in order to reduce food waste throughout the overall food chain, from field to the consumer’s table [12,13,14,15]. However, although several consumer awareness campaigns have already been carried out, it should be noted that international harmonization on what should be considered food waste has not yet been achieved; in fact, there are related, but different, concepts such as waste, loss, leftovers, etc.

Food sustainability is therefore a hot topic nowadays, but it is important to raise the question: is the general population familiar and concerned with this concept? To what extent is there an adequate knowledge on specific dietary sustainability and environmental impact concepts, considering different population age groups? Moreover, which are the general population attitudes and behaviors in order to contribute to food sustainability and therefore, to contribute to challenge the climate change effects?

The very few studies addressing related questions have focused on aspects such as consumers’ willingness-to-pay for sustainable specific products (such as some organic and local foods) [16,17], the possible interaction between millennials and sustainability in the food sector [18], or the knowledge and awareness of general sustainability initiatives among college students [19,20]. Most of these studies support, as a main conclusion, the need to implement a wide range of initiatives aimed at educating consumers about sustainable dietary patterns, thus highlighting the general unawareness of the population, and a missing systematic engagement with formal education on this topic, in the community.

Spain is a country with a deep tradition in terms of Mediterranean dietary habits, and is currently developing different sustainable development strategies [21,22,23,24]; however, at present, there are no representative studies targeting knowledge and perception of the Spanish population on these questions. Based on these issues, the main objective of this study was to evaluate the knowledge on food sustainability and environmental impact concepts, as well as related attitudes and behavior in a representative sample of Spanish adult population. 

## 2. Materials and Methods

### 2.1. Sample and Study Design

We conducted a cross-sectional survey on sociological aspects of food eating habits and food agency in a representative sample of adults (≥18 years old) living in Spain. Results on shopping and cooking habits have been previously published [25,26]. This paper presents the results on food sustainability attitude and knowledge in this same population.

In short, we developed a questionnaire (49 total items) including questions about food sustainability knowledge and perceptions, as well as items on sociodemographic aspects (Appendix A, Table A1), food organization, cooking habits and skills, shopping habits, and food safety. The questions on sustainable eating (Appendix A, Table A2 and Table A3) comprised aspects related to knowledge about sustainability terms and about the environmental impact of different food groups, attitudes towards buying more sustainable products, and linked values to sustainable food shopping (see Appendix A). Questions were based on a thorough analysis of the literature on sociological, dietary habits, and food consumption patterns in Spain. Questionnaire was face-validated by experts from the consulting agency that performed the survey, who had previously participated in surveys on food habits in Spain, jointly with the research team.

Professionally trained surveyors performed the questionnaires, using computer-assisted telephone interviewing (CATI). Interviewees were randomly selected and reached at their private dwellings all over Spain, except for the Autonomous Cities of Ceuta and Melilla in North Africa. 

Phone calls took place between 1:00 and 9:30 p.m., the period when it is more likely to find all dwellers at home. Prior to its definite validation, and in order to guarantee optimum design and applicability, we tested the questionnaire in a small sample. Seventy respondents from 150 telephone calls completed the pre-test in the pilot study. The final survey could be completed within 35 min. Field work was undertaken in September–October 2017, and a total of 55,558 phone calls were made.

Sample size was calculated for a confidence interval of 95.5% a margin of error of 5% and a probability of positive (p) and negative (q) responses p = q = 0.5. from a total universe of 38,314,895 people. Sampling was stratified, setting a minimum sample for the strata where the universe was smaller, with a random selection of individuals to be surveyed. Variables used for stratifying were geographical area (Nielsen areas in Spain), habitat size, sex, and age. No selection bias on educational level or socioeconomical status was considered. Finally, a minimum of 2000 participants was stablished The final distribution presented structural differences as compared to the actual population because of the affixation of the sample by strata. Therefore, a weighting factor according to sex and age was applied in order to save the proportionality of each of the strata of the sample with respect to the actual population under study. Inclusion criteria were to be over 18 years old, to reside permanently in Spain and to give verbal consent for subsequent use and publication of the resulting data in an anonymous way. 

Following the same scheme as census data published by The Spanish National Statistics Institute [27], we categorized the sample in five age groups: 18–30, 31–49, 50–64, 65–74, and >75 years.

The study was conducted in accordance with the Declaration of Helsinki, and all data were collected anonymously and recorded according to the Spanish Organic Law on Protection of Personal Data and guarantee of digital rights 3/2018. Since participants could not be tracked, there was no need for informed written consent. Nonetheless, participants were informed about the aim of the study, and were asked for permission to use and publish the data, before answering the questionnaire.

### 2.2. Statistical Analyses

Analysis was performed excluding missing values due to a low non-response rate. 

Results for categories are reported using frequencies and percentages, and continuous variables are reported using mean ± standard deviation. 

For results given as distributions (knowledge of terms related with sustainability and perceived impact of different food groups on sustainability), differences between groups were evaluated using a chi-square test (*z*-test for multiple comparisons). A two-tailed Student’s *t*-test and ANOVA with a Bonferroni post-hoc test were performed to evaluate the differences between sex and age groups, respectively, depending on whether the variables were continuous or discrete.

Pearson’s correlation was performed between the score given to the importance of buying sustainable food and age, and a paired *t*-test was used to analyze the differences between the score given to the importance of buying sustainable food and that given to the willingness to pay for it.

For all statistical analysis, differences were considered significant at *p* ≤ 0.05. Statistical analyses were performed using SPSS v.24.0 (IBM Corp., Armonk, NY, USA) and Med Calc, v.17.9 (Med Calc Software, Ostend, Belgium).

## 3. Results

Of a total of 56,558 phone calls: 28,252 (49%) failed to contact; 22,339 respondents did not agree to participate in the survey (54% because they did not like to participate in telephone surveys; 31% hung up the phone just after contact; 13% due to “other reasons”, and 2% without statement of any particular motive). From the 5967 respondents willing to participate in the survey: 3347 were excluded because they did not match the inclusion criteria (or were out of quotes at the moment of the contact), and 568 surveys were rejected because of a high number of blank questions (over 50%). Finally, 2052 valid questionnaires were used. The non-response rate to questions after enrollment was very small (1.5% as an average).

The final sampling error was 2.1% for global data, calculated for a confidence interval of 95.5% (CI 95.5%, and p = q = 0.5). By gender: 882 volunteers were men (43%) and 1170 were women (57%), By age group: 266 respondents were 18 to 30 years old (13%), 675 were 31 to 49 years old (33%), 522 were 50 to 64 years old (26%), 316 were 65 to 75 years old (15%), and 273 were older than 75 (13%). Representativeness error for age and sex distribution was 2.2%. Table 1 shows the socioeconomic characteristics of the sample.

### 3.1. General Knowledge on Sustainability and Food Sustainability

**Figure 1:**
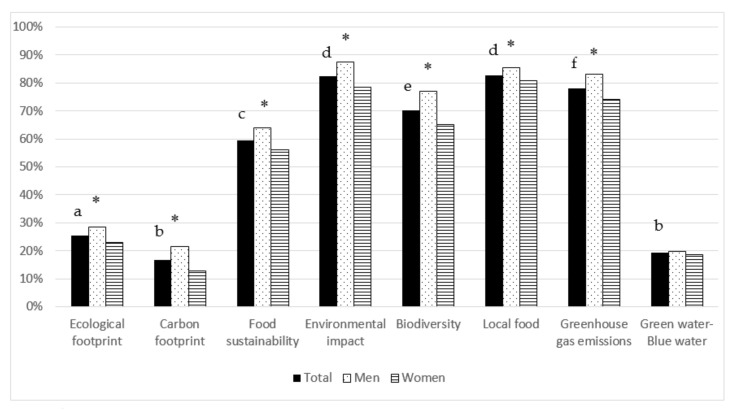
Percentage of Spanish adult population that stated knowing the meaning of different terms related with food sustainability, by gender. Different letter superscripts denote statistical differences (*p* ≤ 0.05) between percentages of people knowing each terms; * shows statistical differences (*p* ≤ 0.001) between men and women. Sample size: 882 men and 1170 women.

Participants were asked about the meaning (“yes/no/have heard the term but does not know what it means”) of different terms related with food sustainability. As shown in Figure 1, most of the terms (5 out of 8) were identified by more than 50% of the population. The best recognized concepts were “environmental impact” and “local food” whilst the less familiar terms were “carbon footprint” and “green water/blue water”. In general, the percentage of respondents that recognized the terms was higher in men than in women, regardless of the age group (Figure 1). Interestingly, the concept of “carbon footprint” was highly unknown by women (87.2% vs. 78.3% of men). 

Table 2 shows the results by age groups. The knowledge about the meaning of the analyzed terms was quite similar in participants aged 18 to 64 years, whereas from 65 years on, the percentage of people stating they know the concepts decreases, for all items. The term “green water/blue water” was highly unknown by all participants regardless of age or sex.

**Figure 2:**
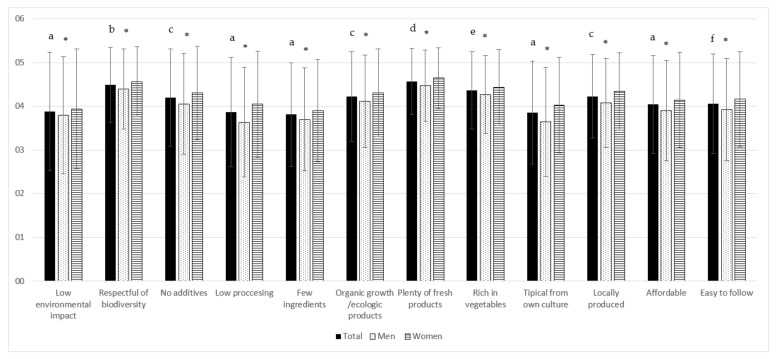
Perceived attributes that define a sustainable diet, in a scale 1–5 (1 = not important at all to 5 = very important) in Spanish adult population, by gender. Different letter superscripts denote statistical differences (*p* ≤ 0.05) between scores given to the different items; * shows statistical differences (*p* ≤ 0.001) between men and women in each item. Sample sizes were *n* = 845 men and *n* = 1107 women; Non respondents = 100 (5%).

Participants were also asked to rate the importance of a list of attributes which can be used to define a sustainable diet, in a scale 1 to 5, being 1 “not important at all” and 5 “very important”. As shown in Figure 2, the surveyed Spanish population perceived a sustainable diet should be “plenty of fresh products” (4.6 ± 0.8), “respectful towards biodiversity” (4.5 ± 0.9) and ”rich in vegetables” (4.4 ± 0.9); while “the cultural aspects of the diet” (3.9 ± 1.2), “be simple (composed of few ingredients)” (3.8 ± 1.2), and, surprisingly, the “environmental impact” (3.9 ± 1.4) showed the lowest scores. The scores given by women were higher than those given by men for all items (Figure 2), regardless of the age group.

Table 3 shows the results on the perception of the importance given to the different attributes that define a sustainable diet, by age groups. The younger group (18–30 years) gave the lowest scores for each item, except for “a sustainable diet is easy to follow”. The most important attribute of a sustainable diet for the participants aged 18–30 years was “diet that maintains biodiversity”, while for the other age groups was “diet plenty of fresh products”.

When linking sustainability to health, 40% of the participants believed that sustainable diet and healthy diet terms were synonymous. This percentage tended to increase with age (35% in the younger group (18–30 years) to 45% in the eldest (>75 years), *p* ≤ 0.0005) and was higher in women (53%) than in men (47%), although not significantly.

**Figure 3:**
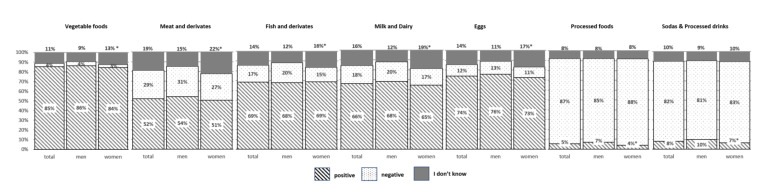
Perceived impact on sustainability of the different food groups in Spanish adult population, by gender. * denotes statistical differences between women and men (*p* ≤ 0.05). Samples size were: Men *n* = 882; women *n* = 1170.

**Figure 4:**
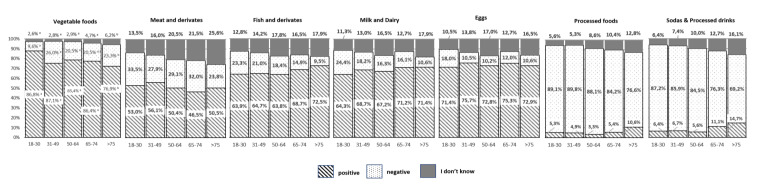
Perceived impact on sustainability of the different food groups in Spanish adult population, by age groups. Different letter superscripts denote statistical differences (*p* ≤ 0.05) between age groups. Samples size were 18–30 years *n* = 266; 31–49 years *n* = 675; 50–64 years *n* = 522; 65–74 years *n* = 316; >75 years *n* = 273.

Figure 3 and Figure 4 show participants’ perception of the impact on sustainability of the different food groups (“positive impact/negative impact/I don’t know”).

A high number of respondents (85%) stated that vegetables contribute positively to food sustainability. Likewise, around 50% of the population think that meat and derivatives contribute positively to food sustainability, whereas around 60% attributes a positive effect to fish, and 70% think so of dairy products. This perception is more common in women (for all age groups) and in people over 50 y, where the “I don’t know” option is also more frequent (*p* ≤ 0.05). On the contrary, participants attribute the main negative impact on sustainability to processed foods (87%) and processed beverages (82%), regardless of gender or age. 

Regarding participants’ perception of the importance of water and its use in food production (Table 4), results showed that water is more needed for vegetable food than for animal food production (*p* ≤ 0.01). Moreover, participants moderately agree with the idea that “enough water for the planet is granted by the natural cycle of water”.

### 3.2. Attitudes to Sustainable Diets

Participants were asked to evaluate how important it is to acquire sustainable food on a scale from 1 to 5, 1 being “not important at all” and 5 “very important”. The total sample’s mean score was 4.22 ± 0.99, and women gave a statistically significant higher score than men (Table 5).

A positive correlation (*p* ≤ 0.0005) was found between the score given to the importance of buying sustainable foods and age. Surprisingly, the youngest participants gave the lowest score to this issue.

When asked to rate their willingness to pay more for a sustainable food, on a similar scale from 1 to 5, 1 being “not willing at all” and 5 “absolutely willing”, the participants rated 3.60 ± 1.2, and accordingly with the response given to the previous question, women were more prone to pay more to afford a sustainable diet than men.

With respect to age differences, the less prone to pay more to move to a sustainable diet were the people older than 75 years (3.40 ± 1.4) while the highest score was given by those between 50–64 years (3.70 ± 1.1) (*p* = 0.006, 50–64 years vs. >75 years).

Finally, scores given to the question “how important is it for you to buy sustainable food?” are significantly higher than those given to the “willingness to pay more for sustainable foods” (*p* ≤ 0.001), in all gender and age groups.

## 4. Discussion

### 4.1. General Knowledge on Sustainability and Food Sustainability

This is the first study in Spain to evaluate adult population’s knowledge of sustainability and environmental concepts, and to assess attitudes and behaviors towards them, developed over a sample size representative at national level. Our results show, globally, that Spanish adults have a positive attitude towards leading a sustainable food choice. However, the concept and attributes, which define a sustainable diet, are still confusing for most part of the population. 

There is still no global consensus on what constitutes healthy diets and sustainable food production, and studies show that few consumers have a high comprehension of the actual sustainable characteristics of food products [28,29]. Most of the published research on the topic shows that the population perception of a sustainable diet is one mainly composed of “foods of plant origin, from organic growth and low processing”. Moreover, most consumers in different countries, when thinking about food sustainability, do not take into account other characteristics such as greenhouse gas emission of food production, water and land footprint or, even less, cultural or social equity [30,31,32]. Our results are mainly in accordance with those assertions, since, when evaluating different characteristics of a sustainable diet, participants gave the highest scores to the concepts related with freshness of the food, amount of vegetables, or respect to biodiversity. Nonetheless, we must point out that the characteristic with the highest score, i.e., “plenty of fresh products”, is a term not only limited to “vegetables”, but also including foods of animal origin, thus providing a potential misunderstanding as participants might be equating “sustainable” to “not processed”.

Although the FAO’s definition of a sustainable diet includes the facts that it should be not only environmentally friendly, but also “culturally acceptable, accessible, economically fair, and affordable; while optimizing natural and human resources”, these aspects are often overlooked, even more by the general population. In the present study, the option “typical from own culture” showed the lowest scores when rating the characteristics of a sustainable diet, and similar results have been found previously. In a comparable study performed in Northern Europe, Van Loo reported that participants associated sustainability with environmental issues in a higher degree than with societal issues [31]. In a similar way, Grunert et al. [33] observed that subjects associate the term of sustainability with items related to the environmental dimension of sustainability (e.g., environmental impact of use of land and water, environmental impact of food production), and less often with items related to the ethical dimension of sustainability (e.g., working conditions and child labor in food production, world’s food supply, etc.). Thus, it seems that most people associate the term “sustainability” to similar concepts, despite belonging to different cultural backgrounds. Communities and cultures that maintain their own traditional food systems are better able to preserve local food specialties with a corresponding crop and animal diversity, together with decreasing food–miles impact on the environment [34]. Consequently, it is important to make people aware that maintaining a traditional diet is good for the health, and for the environment. In the present study, it must be emphasized that, even if the determinant “the cultural aspects of the diet” showed the lowest scores, when evaluating sustainable characteristics, fresh produce is still considered essential to the Spanish shopping basket, according to traditional Mediterranean dietary habits [25]. Therefore, in a certain way, thinking about fresh products may be interpreted as “maintaining the Mediterranean tradition”. 

On the other hand, it is somewhat paradoxical that two items so closely related as “respecting the biodiversity” and “having a low environmental impact” obtained such a different evaluation (the first one achieved one of the highest scores and the second one of the lowest, when asking about the importance given to those terms when defining a sustainable diet). Nevertheless, it must be noted that, although statistical differences exist between the score given to the different determinants, average scores for all items were close to four, meaning that all characteristics were evaluated as “important” when defining a sustainable diet. 

Much research has been conducted on the environmental impacts of various diets, with most of the studies concluding that a diet rich in plant-based foods and with fewer animal source foods is both good for health and the environment. The EAT-Lancet project [5] showed that the global average intake of healthy foods is substantially lower than the reference dietary intake, whereas overconsumption of unhealthy foods is increasing. Using several approaches, researchers found that global adoption of the reference dietary pattern would provide major health and sustainability benefits, including a large reduction in total mortality. 

Our data show that it is quite clear for the surveyed population that vegetables and fruits are the most important food groups for a sustainable diet, and that processed foods and some beverages are not adequate for sustainability. Nevertheless, when asking about the impact on environment of the production of the different types of foods, we found some important misunderstandings. 

It is worth to mention that more than 50% of the population think that meat and derivatives have a positive impact on food sustainability, and nearly 70% state the same for milk and dairy products. However, in general, meat and dairy products are considered the foods with the highest ecological footprint [35]. This lack of awareness of the negative impact of meat consumption on climate change has been previously reported in other studies in different countries [36,37,38,39,40], but never before in Spain. Nowadays it has been demonstrated that production of meat and animal-based products is associated with a high environmental impact, and it is estimated that livestock accounts for approximately 14% of global anthropogenic greenhouse gas emissions [41,42,43]. The recently published Summary Report of the EAT-Lancet Commission [5], states “Transformation to healthy diets by 2050 will require substantial dietary shifts. Global consumption of fruits, vegetables, nuts, and legumes will have to double, and consumption of foods such as red meat and sugar will have to be reduced by more than 50%. A diet rich in plant-based foods and with fewer animal source foods confers both improved health and environmental benefits”. However, meat consumption has undoubtedly cultural and symbolic meanings. People eat meat not only for nutritional reasons, but also for pleasure and to express socio-economic status [36,44]. Most studies show that when talking about changing eating habits to reduce the impact on the environment, the idea of reducing meat consumption is the least accepted by the population [32,45]. In Spain, the intake of meat and meat products is about 47–53 kg/year per capita [46,47]. It is higher in men than in women, and it accounts as the second largest source of energy in the population’s diet, providing 15.2% of total energy intake [48]. 

An increasing amount of literature focuses on fish consumption and diet sustainability [49,50,51,52]. In our research, nearly 70% of the population, regardless of age or gender, stated that fish and derivatives are linked to a sustainable diet. Fish is a good source of proteins, several micronutrients and long-chain fatty acids [53]. On the other hand, although greenhouse gas emission of “fish eaters’ diet” is very similar to that of “vegetarians” [49], some other factors, such as loss of biodiversity, must be taken into account when discussing fish impact on food sustainability. Global seafood consumption has more than doubled in the past 50 years, putting stress on the sustainability of fishing [52]. Improved management of the oceans at a global level is necessary and urgent to ensure that the fishing industry does not have a negative impact on ecosystems, that fish stocks are used responsibly and that global aquaculture production expands in a sustainable and healthy manner [5]. Seafood consumption in Western diets involves a small number of species that are commonly consumed [51], and currently it is estimated that more than 90% of world’s fish stocks are fully exploited, overexploited or collapsed [54]. Whether or not we should decrease the intake of fish to improve sustainability is a matter of debate [55], but moving into conscious purchasing and looking for fish from sustainable fisheries, is well evidence based recommendations if we want to move to more sustainable food habits [56]. The consumption of fish in Spain is about 23 kg/person a year, the second highest world consumption [47]. However, this consumption is slowly decreasing and has fallen by 2.2% in 2018, mainly in the younger groups of the population [46].

The lasts questions in the questionnaire, used in this research, were about beliefs on water sustainability. Water is the largest natural resource, but only 3% of it is freshwater, of which just one-third is accessible for use in agriculture and urban areas. Global agriculture accounts for 70% of freshwater extracted for human use [57]. Moreover, the rising demand for meat and dairy has immediate impacts on land and water use. Approximately half of all cereals globally grown are fed to animals, with the water consumption that this implies [41]. United Nations (UN) studies show that 200 L of water are needed to produce a 200 mL glass of milk, and 2400 L of water to produce a 150 g hamburger [58]. The water footprint of 1kg of bovine meat (18,870 L) is sixty-one times higher than the water footprint of the same amount of vegetables (310 L), and eleven times higher than the water footprint of pasta (1770 L) [35]. The UN’s World Economic and Social Survey in 2011 concluded that intensive livestock production is probably the largest sector-specific source of water pollution [59]. Nevertheless, this information has not reached the general population, since our data show that all participants think that vegetable food production requires a higher amount of water as compared to animal food. Apart from that, the centered value given to the idea “enough water for the planet is granted by the natural cycle of water”, showed a low awareness of the importance of water use. Although Spain has implemented several campaigns to reduce water disposal, due to frequent draughts, our study demonstrates a lack of general knowledge on the use of water for food production. 

### 4.2. Attitudes to Sustainable Diets

When we asked to rate how important it was to buy sustainable food, the samples’ mean score was 4.22 (“very important”); nevertheless, people with a positive attitude towards a certain type of behavior not necessary implement that behavior [60]. The International Food Information Council 2019 study [29], carried on American consumers, showed that only 54% of respondents give importance to environmental sustainability in purchased food products. 

To move to a more sustainable diet, prize may be a barrier. In Spain, some studies have found that the price of organic food can cost up to a 100% more than conventional products [61]. Several studies found that most consumers state [62,63,64] or show [65,66] a general willingness to pay more for organic foods. Some studies show that most consumers may be willing to pay 10% more for more sustainable products [16,67]. The top 10 consumer trends in 2019 study [68] noted that the proportion of those willing to pay more for packaged food and fresh food, which is environmentally conscious or eco-friendly, has risen over the 2015–2017 period. Our results showed higher scores when evaluating the “interest in sustainable food”, as compared to the “willingness to pay for it” but, despite of this, the scores given to the “willingness to pay more” can be considered high (score 3.6/5). In this sense, other studies [66,69,70,71,72] found higher or lower estimates of willingness to pay, depending on the product, the information provided, the respondent profile, and the method used to measure willingness to pay.

In the present study, women and older people were more prone to pay more for sustainable options, which is in line with previous studies in Spain and other European countries [16,73,74]. Illichmann et al. [75]; however, found that men had a higher willingness-to-pay for local and organic food than women.

Even though some published studies failed to find differences between women and men on the attitudes to and knowledge of food sustainability [33,67], our results show a higher interest for sustainability in women as compared to men, which is in agreement with other research [38,45]. At present, cooking and shopping in Spain is still primarily carried out by women [25,26], and therefore food consumption practices are an area where gender roles are still of great significance. This fact may explain women’s higher interest on food sustainability and in consuming sustainably. It can also be taken as an opportunity to increase sustainability interest in this segment of population, and thus empower women as promotors of the change to a more sustainable consumption in the whole family.

In a previous study carried out in Madrid (Spain) metropolitan area, researchers found that although there is a large diversity between the sociodemographic characteristics of the “sustainable buyer”, those were mainly: mature, with older children, or with no children at all [76]. These characteristics clearly match our results, as the population group that give more importance to buying sustainable are those over 50 years. Other studies [33,45,77], also noted that older people were more active adopting sustainable practices. This effect of age may be related not only to ecological values, but also to frugality, experiences and skills learned during less affluent times in the past [78]. The lower interest for sustainable eating among the younger group (18–30 years) is another interesting and somewhat surprising result. This lack of sustainability consciousness may be due to low responsibility of the young group for cooking and shopping at home [25,26], or to a true lesser interest in sustainability. Results from other countries are controversial, while some research show a higher concern for environment and food sustainability in the younger population [18,28,79]; others found similar results to ours [45,77]. In consequence, it remains uncertain whether today’s young people will engage in sustainable consumption through food-related practices in the future.

By definition, a sustainable diet should lead to health [10]. This idea is what 40% of participants in this research believe since they stated that sustainable diet and healthy diet are synonymous. Similar results were found by others [30,80,81]. Moreover, some data obtained by our research team show that the most important factor the Spanish population take into account when purchasing food is “looking for healthier food” (92,6%) [82]. The affirmation that sustainable diet and healthy diet are synonymous increased with age and was more frequent in women than in men. Similarly, Barone [30] observed that middle-aged women and adults might be more prone to associate sustainability with health, due to the associations between organic foods, natural foods, and health, the latter being less worrisome for young people and men. The synergy of sustainability—health may be useful as a communication strategy to promote the shift toward more sustainable diets, as also proposed by other authors [31,83,84].

Ensuring sustainable consumption is one of the 17 Sustainable Development Goals of the 2030 Agenda for Sustainable Development adopted by world leaders in September 2015 [85]. Food production and consumption are major issues in the politics of sustainability, since they are linked to environmental pollution, greenhouse gases emission, water waste, soil degradation, and loss of biodiversity [5,43,79,86]. Achieving the Development Agenda will be impossible or time-costly, without changing food consumption habits of the population.

Consumers, by their choices, have a major role in making food chain more sustainable as they may influence the way that food is being produced and managed. Changing individual’s food consumption patterns can have a deep effect in mitigating greenhouse gases emissions [87,88]. In developed countries, consumer’s food choices are very complex and do not only depend on sociodemographic characteristics, but also on values and motivation. “Political consumption” is a recent term which has been defined as “a form of consumption that involves social, cultural, animal related, and environmental concerns that go beyond the immediate self-interests of the individual consumer or household” [89]. “Political consumers” choose their products and services based on the politics of the product, more than on private virtues, such as price, taste, and healthiness of food [77]. To become political consumers, people need motivation and adequate information on the social and environmental consequences of products. Knowing the attitudes towards and knowledge on food sustainability of the general population is, consequently, a key point to design interventions than can move consumers’ purchasing to more conscious and sustainable options, since it has been found that people’s willingness to adopt climate-friendly behaviors increases when they know more about effective climate-friendly actions [90].

Data show that a great proportion of Spanish population links health and sustainability. Research has demonstrated the health consciousness influences on purchasing habits [91,92]. Health consciousness can result in a deeper interest in knowing the characteristics of a sustainable diet, as well as linking sustainability to health can improve the shift to a more environmentally friendly food purchasing. Some authors demonstrated that participants might judge the environmental impact of a food product or behavior more favorably if the product or behavior is also considered healthy [84]. The women in this study showed a greater interest in food sustainability, in general. Thus, intervention targeted to women have a higher potential to be successful, since women are more health conscious and show a greater interest in sustainable and healthy food [93], together with them being the member of the family in charge of the majority of food purchasing and cooking [25,26]. Well-informed and motivated women can be the impulse to the sustainable shift of the families and general population.

The difference in results between age groups show that, even though youth movements to protect the planet are increasing worldwide, the lack of interest of the younger generations for food habits, in general, result in a lesser knowledge of the impact of food in sustainability. More strength should be addressed in designing policies promoting healthy and sustainable cooking and food consumption habits, from the school. 

To shift into a more sustainable eating population requires information and motivation. Policies should be launched to clarify how our food consumption affects the future of the planet, and to inform and empower people to make the right diet choice for the present and the future. A recent paper by the FAO and the Food Climate Research Network [94] found that 83 countries in the world—of the total 215—produce official dietary guidelines, and that only around nine of those—seven of them in Europe—include sustainability criteria. To elaborate sustainable dietary guidelines, accompanied by credible implementation strategies [43,95,96], is fundamental if we want people to support a healthy and sustainable diet. The recently published Food and Dietary Guidelines for Spanish population, developed by the Spanish Society of Community Nutrition (SENC) [97], include tips for sustainable choice and waste reduction. Further research should be done to evaluate their impact on people’s food behavior.

The big sample size and its representativeness is an important strength of our study. Despite that some part of the population might be under-represented because calls were done only to house phones. The reason for that approach is that we were intending to improve collaboration, since the relative long duration of the questionnaire, and the nature of the questions required a great degree of attention and dedication, which is not easy to achieve when the individuals are at work or doing other activities. Moreover, data were self-reported, and therefore potentially exposed to memory and social desirability bias. Other limitations include the initial low participation rate, although once contacted, the participant’s involvement in the survey was good, and the fact that validation of the survey was only based on face validity.

## 5. Conclusions

In conclusion, this study is the first one to show data about Spanish adult population’s attitudes and knowledge on food sustainability at a national level, and from a holistic point of view. The results suggest that Spanish adult population show a high interest in eating sustainable and healthy, but this idea is not enough supported on evidence-based knowledge and embraces some important misconceptions on the characteristics of a sustainable diet. Therefore, there is an urgent need of interventions to help people understand how food impacts on the environment.

## Figures and Tables

**Table 1:** Percentage of surveyed population according to level of education, employment status, and household income.

Categories	Level of Education
Cannot read or write	0.1
Less than primary school	6.3
Primary school	18.1
Secondary school	22.2
Technical education	12.2
University studies (Diploma)	11.6
University studies (Degree, Masters, PhD)	28.6
DK/DA	0.9
	**Employment Status**
Employee	47.1
Domestic worker (non-paid)	7.5
Unemployed	12.4
Student	8.2
Retired	24.5
DK/DA	0.3
	**Household Income**
<1000 euros	16.9
1001–2000 euros	30.1
2001–3000 euros	18.5
3001–4000 euros	7.6
>4000 euros	4.8
DK/DA	22.1

Note: DK/DA: do not know/do not answer.

**Table 2:** Percentage of Spanish adult population that stated knowing the meaning of different terms related with food sustainability, by age groups.

Food-Sustainability Related Term	Age Groups (Years)				
	18–30 years *n* = 266	31–49 years *n* = 675	50–64 years *n* = 522	65–74 years *n* = 316	>75 years *n* = 273
**Ecological footprint**	36.0 ^a^	33.6 ^b^	30.1 ^b^	18.0 ^c^	17.0 ^c^
**Carbon footprint**	25.2 ^a^	25.9 ^a^	24.4 ^a^	13.7 ^b^	10.0 ^b^
**Food sustainability**	64.0 ^a,b^	74.5 ^b^	67.9 ^a^	56.8 ^c^	35.0 ^d^
**Environmental impact**	92.8 ^a,b^	92.3 ^b^	89.0 ^a^	83.5 ^c^	69.0 ^d^
**Biodiversity**	82.0 ^a^	83.2 ^a^	82.9 ^a^	74.8 ^b^	43.0 ^c^
**Local food**	82.0 ^a^	90.2 ^a^	88.6 ^a^	84.2 ^b^	69.0 ^c^
**Greenhouse gas emissions**	89.2 ^a^	87.8 ^a^	90.2 ^a^	77.7 ^b^	54.0 ^c^
**Green water–blue water**	14.4 ^a^	19.9 ^a^	19.1 ^a^	28.1 ^a^	16.0 ^a^

Note: Different letter superscripts denote statistical differences (*p* ≤ 0.05) between percentages of people knowing each term in each age group.

**Table 3:** Perceived attributes that define a sustainable diet, in a scale 1–5 (1 = not important at all to 5 = very important) in Spanish adult population, by age groups.

Attributes	18–30 years *n* = 258	31–49 years *n* = 658	50–64 years *n* = 496	65–74 years *n* = 297	>75 years *n* = 243
Low environmental impact	3.85 ± 1.2 ^a^	3.88 ± 1.4 ^a^	3.90 ± 1.4 ^a^	3.93 ± 1.3 ^a^	3.80 ± 1.4 ^a^
Respect towards biodiversity	4.37 ± 0.8 ^a,c^	4.52 ± 0.8 ^a,b^	4.60 ± 0.8 ^b^	4.57 ± 0.7 ^a,b^	4.21 ± 1.1 ^c^
No additives	3.95 ± 1.0 ^a^	4.23 ± 0.1 ^b^	4.30 ± 0.1 ^b^	4.27 ± 1.2 ^b^	4.06 ± 1.4 ^a,b^
Low processing	3.66 ± 1.1 ^a^	3.94 ± 1.2 ^b^	3.97 ± 1.2 ^b^	3.88 ± 1.3 ^a,b^	3.65 ± 1.4 ^a^
Few ingredients	3.35 ± 1.1 ^a^	3.67 ± 1.2 ^b^	3.93 ± 1.1 ^c^	4.13 ± 1.0 ^c^	4.08 ± 1.1 ^c^
Organic growth/ecologic products	4.07 ± 1.0 ^a^	4.25 ± 1.0 ^a^	4.26 ± 1.0 ^a^	4.28 ± 1.0 ^a^	4.16 ± 1.0 ^a^
Plenty of fresh products	4.30 ± 0.9 ^a^	4.56 ± 0.7 ^b^	4.65 ± 0.7 ^b,c^	4.70 ± 0.6 ^c^	4.57 ± 0.8 ^b,c^
Rich in vegetables	4.09 ± 0.9 ^a^	4.32 ± 0.9 ^b^	4.43 ± 0.9 ^b,c^	4.55 ± 0.7 ^c^	4.40 ± 1.0 ^b,c^
Typical from own culture	3.51 ± 1.2 ^a^	3.68 ± 1.2 ^a^	3.94 ± 1.2 ^b^	4.16 ± 1.0 ^b,c^	4.21 ± 1.0 ^c^
Locally produced	3.80 ± 1.1 ^a^	4.1 ± 0.9 ^b^	4.38 ± 0.9 ^c^	4.42 ± 0.9 ^c^	4.33 ± 0.9 ^b,c^
Affordable	3.69 ± 1.2 ^a^	3.84 ± 1.2 ^a^	4.20 ± 1.0 ^b^	4.39 ± 0.9 ^b^	4.21 ± 1.0 ^b^
Easy to follow	4.04 ± 1.2 ^a^	3.65 ± 1.2 ^b^	3.96 ± 1.1 ^b,c^	4.13 ± 0.9 ^c^	4.35 ± 1.0 ^c^

Note: Different letter superscripts denote statistical differences (*p* ≤ 0.05) between scores given to different items by the different age groups. Non respondents = 100 (5%).

**Table 4:** Perceived importance of water-use in food production, in a scale 1–5 (1 = totally disagree; 5 = totally agree), in adult Spanish population, by gender and age groups.

Group	1. Enough Water for the Planet is Granted by the Natural Cycle of Water	2.The Foods Requiring a Greater Expenditure of Water are of Animal Origin	3. The Foods Requiring a Greater Expenditure of Water are of Vegetable Origin	*p* (2 vs. 3)
Total	3.41 ± 1.45	3.14 ± 1.27	3.88 ± 1.19	0.0005
Gender				
Men	3.44 ± 1.43	3.79 ± 1.23	3.79 ± 1.23	0.0005
Women	3.39 ± 1.46 *	3.95 ± 1.16 *	3.95 ± 1.16 *	
Age				
18–30 years	3.47 ± 1.32 ^a,b^	3.58 ± 1.18 ^a^	3.58 ± 1.18 ^a^	0.003
31–49 years	3.36 ± 1.47 ^a,b^	3.80 ± 1.19 ^a,b^	3.80 ± 1.19 ^a,b^	0.0005
50–64 years	3.33 ± 1.47 ^a^	3.92 ± 1.26 ^b,c^	3.92 ± 1.26 ^b,c^	
65–75 years	3.44 ± 1.43 ^a,b^	4.10 ± 1.02 ^c^	4.10 ± 1.02 ^c^	
>75 years	3.67 ± 1.38 ^b^	3.28 ± 1.33 ^c^	4.09 ± 1.18 ^c^	

* Denotes statistical differences (*p* ≤ 0.05 between men and women. The samples sizes were men *N* = 782 and women *N* = 1011. Different letter superscripts show statistical differences (*p* ≤ 0.05) between age groups (columns). Sample sizes: 18–30 years *n* = 245; 31–49 years *n* = 594; 50–64 years *n* = 453; 65–74 years *n* = 273; >75 years *n* = 228. *p* (2 vs. 3) shows statistical differences between questions 2 and 3. No respondents: 9% of total population did not answer question 1 (*n* = 185) and a 12% did not answer questions 2 and 3 (*n* = 259).

**Table 5:** Importance and willingness to pay for a sustainable diet, by score, according to age and sex.

Group	How Important Is It for You to Buy Sustainable Foods? *	To What Extent Are You Willing to Pay More for Food That is Produced Sustainably? **	*p*
**Total**	4.22 ± 0.99	3.59 ± 1.12	0.0005
Gender			
Men	4.08 ± 1.09 ^a^	3.47 ± 1.12 ^a^	0.0005
Women	4.33 ± 0.90 ^a^	3.67 ± 1.14 ^b^	
Age			
18–30 years	4.03 ± 1.10 ^a^	3.50 ± 0.91 ^a,b^	0.0005
31–49 years	4.23 ± 0.9 ^b^	3.55 ± 1.14 ^a,b^	
50–64 years	4.23 ± 0.98 ^a,b^	3.72 ± 1.14 ^b^	
65–75 years	4.31 ± 1.03 ^b^	3.68 ± 1.25 ^a,b^	
>75 years	4.25 ± 1.14 ^a,b^	3.40 ± 1.39 ^a^	

Different letter superscripts show statistical differences (*p* ≤ 0.05) between scores given to different items by gender groups and by age groups (significance between rods). Sample sizes were: Men *n* = 834; Women *n*= 1078 and by age group: 18–30 years *n* = 256; 31–49 *n*= 653; 50–64 years *n*= 483; 65–74 years *n*= 295 and > 75 years *n*= 225; Non respondents *n*= 140 (7%).* Scale: 1 “not important at all” to 5 “very important”. ** Scale: 1 “not willing at all” to 5 “absolutely willing”.

## Appendix A. Questionnaire on Food Sustainability Knowledge and Attitudes to Sustainable Eating

**Table A1:** Sociodemographic Data.

**Q.1. Gender:**	code	**Q.2. How old are you? Age:**	code	**Q.3. Where do you live?** **Province: ________________** **City/Village: _____________** **Habitat size**	code
Male	1	18–30 years	1	<2000 inhabitants	1
Female	2	31–49 years	2	2001–10,000 inhabitants	2
		50–64 years	3	10,001–10,000 inhabitants	3
		65–75 years	4	100,001–500,000 inhabitants	4
		>75 years	5	>500,000 inhabitants	5
**Q.4. Nationality**		code	**Q.5. Employment Status: Are you currently…?**	code	**Q.5.1. How long have you been unemployed?**
Spanish		1	Employee	1	______________________
Other: ___________		2	Domestic worker (non-paid)	2	
			Unemployed(Go to Question Q.5.1)	3	
			Student	4	
			Retired	5	
			DK/DA	6	
**Q.6. What is your highest level of studies?**			Code	**Q.7. Household income**	Code
Cannot read or write			1	<1000 euros	1
Less than primary school			2	1001–2000 euros	2
Primary school			3	2001–3000 euros	3
Secondary school			4	3001–4000 euros	4
Technical education			5	>4000 euros	5
University studies (Diploma)			6	DK/DA	6
University studies (Degree, Masters, PhD)			7		
DK/DA			8		

**Table A2:** Knowledge on Sustainability and Food Sustainability.

**Q.8. Do you know the following concepts?**						
**Concepts**	Yes		No	Have heard the term but does not know what it means		
Ecological footprint	1		2	3		
Carbon footprint	1		2	3		
Food sustainability	1		2	3		
Environmental impact	1		2	3		
Biodiversity	1		2	3		
Local food	1		2	3		
Greenhouse gas emissions	1		2	3		
Green water–Blue water	1		2	3		
**Q.9. From 1 to 5, to what extent do you consider that each of the following aspects contributes to a sustainable diet? Being 1: “Not important at all” and 5: “Very important”**						
**Aspects**	Not Important at All	Of Little Importance	Moderately Important	Important	Very Important	DK/DA
Low environmental impact	1	2	3	4	5	0
Respectful of biodiversity	1	2	3	4	5	0
No additives	1	2	3	4	5	0
Low processing	1	2	3	4	5	0
Few ingredients	1	2	3	4	5	0
Organic growth/ecologic products	1	2	3	4	5	0
Plenty of fresh products	1	2	3	4	5	0
Rich in vegetables	1	2	3	4	5	0
Typical from own culture	1	2	3	4	5	0
Locally produced	1	2	3	4	5	0
Affordable	1	2	3	4	5	0
Easy to follow	1	2	3	4	5	0
**Q.10. Do you believe that sustainable diet and healthy diet terms are synonymous?**			**Code**			
Yes			1			
Are similar concepts, but not the same			2			
No			4			
DK/DA			0			
**Q.11. Please give your opinion on the contribution of the following foods to the sustainability of the planet (less damage)**						
			Positive Impact	Negative Impact	I Don’t Know (DK/DA)	
Vegetable foods			1	2	3	
Meat and derivates			1	2	3	
Fish, shellfish, and derivates			1	2	3	
Milk and Dairy			1	2	3	
Eggs			1	2	3	
Processed foods			1	2	3	
Sodas and processed drinks			1	2	3	
**Q.12. From 1 to 5, indicate to what extent you agree with the following statements related to water and its use in food production. Being 1: “Do not agree” and 5 “Completely agree”.**						
	Do not agree	Agree a Little	Mostly Agree	Agree	Completely Agree	DK/DA
Enough water for the planet is granted by the natural cycle of water	1	2	3	4	5	0
The foods requiring a greater expenditure of water are of animal origin	1	2	3	4	5	0
The foods requiring a greater expenditure of water are of vegetable origin	1	2	3	4	5	0

**Table A3:** Attitudes to Sustainable Diets.

**Q.13. From 1 to 5: How important is it for you that the products you consume are produced in a sustainable way? Being 1: “Not important at all” and 5 “Very important”.**					
Not Important at All	Of Little Importance	Moderately Important	Important	Very Important	DK/DA
1	2	3	4	5	0
1	2	3	4	5	0
1	2	3	4	5	0
**Q.14. From 1 to 5: To what extent are you willing to pay more money for food and drink products that are produced in a sustainable way? Being 1: “Not at all” and 5 “Willing”.**					
Not at All	Unwilling	Moderately Willing	Quite Willing	Willing	DK/DA
1	2	3	4	5	0
1	2	3	4	5	0
1	2	3	4	5	0
